# Ethical issues of informed consent in malaria research proposals submitted to a research ethics committee in Thailand: a retrospective document review

**DOI:** 10.1186/s12910-017-0210-0

**Published:** 2017-08-14

**Authors:** Pornpimon Adams, Sukanya Prakobtham, Chanthima Limpattaracharoen, Sumeth Suebtrakul, Pitchapa Vutikes, Srisin Khusmith, Polrat Wilairatana, Paul Adams, Jaranit Kaewkungwal

**Affiliations:** 1 Office of Research Services, Faculty of Tropical Medicine, Mahidol University, Bangkok, Thailand; 2 Department of Microbiology and Immunology, Faculty of Tropical Medicine, Mahidol University, Bangkok, Thailand; 3 Department of Clinical Tropical Medicine, Faculty of Tropical Medicine, Mahidol University, Bangkok, Thailand; 4 Department of Tropical Hygiene, Faculty of Tropical Medicine, Mahidol University, Ratchawithi Road, Bangkok, 10400 Thailand

**Keywords:** Informed consent process, Ethics, Institutional review board (IRB), Malaria research proposal

## Abstract

**Background:**

The informed-consent process should be one of meaningful information exchange between researchers and study participants. One of the responsibilities of research ethics committees is to oversee appropriate informed consent. The committee must consider various matters before deciding whether the process is appropriate, including the adequacy and completeness of the written information provided to study participants, and the process of obtaining informed consent.

This study aimed to identify, quantitatively and qualitatively, consent-related issues in different types of malaria proposals submitted to the Faculty of Tropical Medicine, Ethics Committee.

**Methods:**

This study reviewed proposal documentation submitted to two panels of the Ethics Committee of the Faculty of Tropical Medicine, Mahidol University, from 2011 to 2015. The documentation included proposals, notifications to researchers of review outcomes and ethical issues of concern to committee members. Each element of the informed-consent process was identified and analyzed by study classification, design, and specimen use, including whether the study involved a vulnerable population. Summative content analysis was used to analyze patterns of common issues raised in reviews.

**Results:**

Of the 112 proposals reviewed, 63 required an informed consent process. All researchers proposed communicating with their study participants; however, about two-thirds needed to improve their explanations of study procedures (study activities and specimen/data-collection process) to participants. About 40% of the proposals attracted comments on informed-consent process elements--risk and discomfort, vulnerable status, and compensation. Studies that planned to collect or use new/linked specimens raised more issues around informed consent than studies using linked data/records. Studies that involved vulnerable populations raised more issues than those that did not. The committee usually asked researchers to clarify, elaborate, revise, or paraphrase the consent process elements that were considered to involve inadequate information exchange between researcher and study participant.

**Conclusions:**

This study aimed to describe lessons for malaria researchers about common informed-consent process issues in different types of malaria proposals. The information and analysis of informed-consent elements should assist the preparation of malaria-research proposals.

## Background

*The Declaration of Helsinki Ethical Principles for Medical Research Involving Human Subjects* [1] makes clear that although the goal of medical research is to generate new knowledge, this can never take precedence over the rights and interests of individuals directly or indirectly participating in a research study. It goes on to state that the lives, health, dignity, and integrity of human research subjects must be protected, along with their right to self-determination, privacy, and confidentiality, even when they have agreed to participate [1]. The many definitions of “informed consent” reflect the ethical, legal, and practical conceptions of this term [1–9]. All, however, are based on the principle of an “informed and free decision”. Directive 2001/20/EC of the European Parliament and of the Council [8] affirms that *“Informed Consent is the decision, which must be written, dated, and signed, to take part in a clinical trial, taken freely after being duly informed of its nature, significance, implications and risks and appropriately documented, by any person capable of giving consent”*. The Consumers’ Health Forum of Australia [9] refers to the National Health and Medical Research Council (NHMRC) guidelines and holds that informed consent must be voluntary, and the person concerned must have been given sufficient information for an informed decision to be made. Like several other medical research organizations, the U.S. Food and Drug Administration (FDA) [6] describes the informed consent process (ICP) as involving more than a signature on a form; it is a process of meaningful information exchange or communication between the study investigator(s) and study participants. The key concepts of an informed consent process distilled from the literature indicate that study participants must (a) understand the information provided, (b) not feel that they are being pressured or coerced into making a decision, and (c) have time to consider their options [7–11].

The principle of informed consent, in both biomedical research and clinical experiments, requires that scientists and investigators behave ethically at all times in treating study participants as autonomous beings, ensuring they are treated with justice, beneficence and respect. However, this does not always occur [12–14]. Unethical behavior and the loss of participant rights are possible when researchers and/or participants neglect the importance of the process. Although basic ethical principles are known by most researchers, there are still covert barriers to understanding the ICP (e.g., language barriers, religious influences, and false expectations) which might lead to ineffective communication between researchers and participants [12]. Effective communication reportedly influences the ICP, especially as it relates to patient-centered care and safety [13]. Potential barriers to study participants’ understanding include ineffective communication, a lack of basic information on the consent form, lack of shared decision-making, lack of consideration of participant health literacy level, and lack of consideration of cultural issues [13]. Obstacles to an ethical and effective ICP for medical research, particularly among ethnic minority patients, include factors such as (a) lack of comprehension and capacity due to overt linguistic barriers and limitations in translating some biomedical terms; (b) the literacy levels of study participants; (c) the notion of autonomy and disclosure of information, which varies considerably between cultural groups; (d) familiarity with research methodology, particularly for groups living in isolation from mainstream culture; and (e) power relationships and feelings of obligation between investigator and study participant [14].

The importance and integrity of informed consent is based on a partnership between the investigator and study participants, in the context that research is a privilege, not a right, and the goal is not merely to have a signature on the consent form, but a shared understanding and decision-making process [15]. It was suggested in the literature that a partnership can occur when a study is presented to study participants in a clear way [15]; however, many may argue that the partnership between equals might be an ideal, but ICP is rather a matter of protecting participants, particularly those who are vulnerable. Again, one should bear in mind that the ability to accept or decline access to private information is a core right of the person; and thus protection of research participants is generally accepted as a right. International guidelines on biomedical research involving human subjects state that one of the responsibilities of research ethics committees (RECs) is to oversee the conduct of appropriate ICPs. The REC must consider various matters before deciding whether the proposed ICP is acceptable, including the adequacy and completeness of the written information to be provided to the study participants, and the process for obtaining informed consent [16]. A critical role and responsibility of the REC (Institutional Review Board (IRB) in US FDA regulations) in ensuring the adequacy of the ICP is to review all of the materials used in it, including the recruitment materials and information provided, and the informed consent document (for example, a chart explaining what to expect at each study visit or a document explaining the costs to subjects) [6]. The REC must ensure that the ICP minimizes the possibility of coercion and undue influence. Committees have the authority to require that information to be given to study participants is meaningful and protects their rights and welfare. It also has both the authority and responsibility to require that the information provided is worded comprehensibly and appropriately, and uses standardized language or format in particular parts of the consent forms (for example, for those elements dealing with confidentiality, compensation, and the voluntary nature of participation). In its review of a clinical investigation, the REC can decide not to approve a study if the ICP does not fit with informed consent regulations. Should there be any subsequent changes to the ICP, the REC should ensure that there is a way to identify such revision(s).

A few studies of ethical issues in malaria research have been reported. This type of research is mainly conducted in areas of developing countries where malaria is endemic. Most studies reported that ethical issues were especially common in clinical trials, including in the ethical review process, standard of care, incentives and reimbursement, and insurance and indemnity [17–20]. A previous study stratified the ethical issues raised in a broad spectrum of research proposals submitted to the Faculty of Tropical Medicine Ethics Committee (FTM-EC) at Mahidol University [20]. The findings of the study described ethical considerations in the review process, but did not focus on ICP issues. The ICP is particularly critical in studies that use new or linked specimens or data in human research subjects, and deficits in the ICP are often identified in a REC review [20–22]. This study focused particularly on ICP in malaria research, since (a) about one-third of proposals submitted to FTM-EC were related to malaria and FTM has been one of the institutes contributing significantly to global research publication on malaria, and (b) ICP in malaria research is crucial as malaria studies are generally conducted among the vulnerable, including children, ethnic groups, refugee and cross-border populations, who are mostly illiterate and reside in remote, limited healthcare-access and/or low-resource settings. With such study characteristics, when reviewing proposals, researchers sometimes argued that the FTM-EC committees over-protected study participants. As malaria research is generally conducted in similar settings to Thailand, malaria researchers elsewhere might benefit from a list of common problems in the ICP elements observed by the FTM-EC. The main purpose of this study is thus to identify, quantitatively and qualitatively, specific defective or incomplete ICP elements that are often presented in the proposals and raised by the ethical review boards. It aims particularly to clarify some misunderstandings and/or arguments of malaria researchers about ICP issues in different types of malaria proposals submitted to and reviewed by the FTM-EC from 2011 to 2015.

## Methods

### Study site and settings

The Faculty of Tropical Medicine, Mahidol University, is an institute renowned for malaria research studies. Based on malaria publication data from 2011 to 2015, using the *SciVal-Worldwide* database (*http://www.elsevier.com/online-tools/research-intelligence/products-and-services/scival* *),* the number of research studies from Mahidol University (Thailand) ranked # 4 (516 papers). Of these, the Faculty of Tropical Medicine (FTM), a Mahidol University faculty, contributed 85.85% (443 papers), correlating to a ranking of #7 if counted as an independent institute. To facilitate research, the Office of Research Services (ORS) provides administrative services to the Faculty’s research community, and secretariat services to the FTM-EC, including managing the operation of Ethics Committee meetings. FTM-EC has been continuously registered with the Federal-wide Assurance (FWA) of the US Office for Human Research Protections (OHRP) since 2002. The committee has two panels, with different members. The clinical panel reviews clinical research studies involving clinical interventions with human research subjects, and the non-clinical panel reviews other types of biomedical study, including research conducted in clinical settings where no clinical intervention was applied, epidemiological studies, and studies that use stored specimens or secondary data. All malaria study proposals submitted to FTM-EC fall into one of these two categories and are reviewed by the relevant panel.

### Elements of the ICP

International standard guidelines [2, 6–8, 11] list the elements that should be considered as part of the ICP. The classic ICH-GCP guideline [11] suggests key words for inclusion in written information provided to study participants, including explanations: (a) that the trial involves research; (b) the purpose of the trial; (c) the treatment(s) and the probability for random assignment to each treatment (if any); (d) the procedures to be followed; (e) the responsibilities of the study participants; (f) the reasonably foreseeable risks or inconveniences; (g) the benefits that can reasonably be expected; (h) the alternative procedure(s) (if any); (i) the compensation, anticipated expenses, and/or payment in the event of trial-related injury; (j) the voluntary nature of participation; and (k) the roles of the monitor(s)/auditor(s)/IRB/REC in the study. Other guidelines identify similar elements.

The US FDA guideline [6] expands on some elements, for example, (a) the ICP should cover not only information presented in documents such as the protocol, but also the investigator’s brochure, package labeling, and previous research study reports; (b) the consent document should provide the name of a specific office or person and the telephone number to contact for answers to questions about the research participants’ rights, research-related injury, and the research study itself; (c) that research participants may choose to discontinue participation in the study at any time without losing the benefits to which they are entitled.

The Royal College of Nursing [23] suggested that informed consent in health and social care research should also ensure potential participants understand the purpose of the research, the duration of their participation, the stakeholders involved in the research, how the data will be managed and used, how long and where the data will be stored, the purpose of the consent form, and that the research has been approved by the relevant REC(s).

The literature suggests [24–26] that consent should be thought of as a process and not only as a document. This process, which could be applied in both conventional clinical practice and research studies, requires disclosure of information and its implications to a person with capacity who understands and then voluntarily decides on whether to participate. In this study, the REC review of the ICP in a proposal is not limited to the documentation of consent (i.e., Participant Information Sheet (PIS) and Informed Consent/Assent Forms (ICF/IAF)), but also includes the context of the proposal and the study procedures relevant to the research participants (e.g., recruitment, treatment and related activities). To summarize the key elements of the ICP for this study, issues raised by the REC were classified into purpose of the study, duration of the study, procedure for data/specimen collection, potential risk and discomfort, method for minimizing risk, potential benefit, confidentiality, the voluntary nature of participation, right to withdraw from the study, managing vulnerable populations, coverage of costs (if any), rationale and justification for compensation, emergency contact name/telephone number(s), and notifiable body if protocol compliance is breached.

### Classification of malaria research studies

WHO guidance for ethical review suggests that RECs may review different types of research studies, and should therefore be familiar with the different methodologies and ethical considerations that apply to each type of proposal [2]. Different types of study include, but are not limited to, clinical trials, epidemiological research, social science research, research on medical records or other personal information, research on stored samples, health systems research, and implementation research. The Medical Research Council, in its Ethics Guides, addresses the uses of information in medical research, including the collection of new information as part of clinical trials or other patient-based research, and the use of information from general practice or existing hospital records [27, 28]. It also notes that confidentiality concerns more often arise in epidemiological or survey work, when information is extracted from medical records without the person’s knowledge or consent. The use of specimens or samples in conjunction with personal data also raises special issues. Several guidelines [27–30] classify and define different types of data collected and used in a study, including anonymized data (linked and unlinked anonymized data, coded data, confidential information, personal information, and sensitive information).

Traditional standards of informed consent require that research participants should be provided with sufficient information about the research activities and the data to be collected from them before making a decision about their participation. The use of left-over or archived specimens is typically thought to involve minimal risk, but it must be made clear during the ICP [29]. The use of specimens and data beyond the purpose originally described in the consent form is another issue of concern to RECs [30].

In this study, the classification is based on research study design, consisting of (1) clinical (investigational new drug, IND) trials, (2) biomedical studies, (3) laboratory studies, and (4) epidemiological/social science studies. For classifications based on collection and use of specimens/data, the study procedure was classified as either collecting new specimens, using archived specimens (linked), reviewing existing medical records/charts (linked), administering questionnaires or interviews to collect new information, or using specimens/medical records (unlinked). The two major categories are (1) collection or use of new or linked specimens/data, and (2) use of unlinked specimens/data. The study population was divided into two categories: (1) studies involving a vulnerable population (i.e., ethnic minority, children, pregnant women, older or unconscious patients), and (2) those not involving any vulnerable populations.

### Sources of information and data analysis

During 2011–2015, 124 malaria research proposals were submitted to the FTM-EC. After excluding six new proposals pending review and three non-approved protocols (due to concerns about very high risk and/or insufficient safety measures in the study design), three protocols withdrawn by the investigators or FTM-EC (due to incomplete documentation or lack of support from sponsor), 112 proposals were either approved or deferred. Of the remaining 112 proposals, 63 required an informed consent process, comprising 11 (17.4%) clinical (IND) trials, 17 (27.0%) biomedical/clinical studies, 22 (35.0%) laboratory studies, and 13 (20.6%) epidemiological/social science studies. For classification based on collection and use of specimens/data, the studies included 52 (82.5%) collecting new specimens, 2 (3.2%) using archived linked-specimens, 2 (3.2%) reviewing existing linked-medical records/charts, 7 (11.1%) administering questionnaires or interviews and 0 (0.0%) using unlinked specimens/medical records.

This study was based on a documentation review. The documentation included the original proposals and notifications to researchers, informing them of review outcomes and ethical issues of concern to FTM-EC members. The notifications to researchers included detailed information from the FTM-EC review about protocol content, PIS, ICF/IAF, and other documents or materials (e.g., advertisements and handouts). Information was extracted by personnel working in the ethics section of the ORS. To avoid bias in the content analysis [31], three ORS employees, acting as data extractors, were assigned to identify the ethical considerations, classified by the ICP elements in the notification to researchers. The employees are in charge of proposal submission and managing notifications. They attend the full board meetings but are non-voting members of the FTM-EC. The checklist of ICP elements was used as a tool for data collection and quantitative analysis, and each data extractor independently read all notifications and related documents. Each element was identified and counted to determine whether it appeared in the notification and matched with the related text described in the original proposals. The three checklists produced as a result were cross validated. The analysis of ICP elements was shown by study classification, study design, specimen use, and whether they involved a vulnerable population.

To identify the common ICP issues in FTM-EC reviews, proposals and notifications to researchers were subjected to content analysis. The purpose of this is to describe and classify written material into identified categories with similar meanings [32]. The categories therefore represent either explicit or inferred communication between the FTM-EC and the researchers submitting the proposals. This study employed summative content analysis, rather than conventional or directed methods [33]. In conventional content analysis, categories are derived from data during the data analysis, to gain a richer understanding of a phenomenon. Directed content analysis uses existing theory or prior research to develop the initial coding scheme before data analysis [33]. The summative approach analyzes patterns (often as single words) in relation to the particular content rather than analyzing the data as a whole, and so was considered a good fit with this study [33, 34]. Summative analysis offers the opportunity to consider data representation while involving a team of co-researchers in the analysis process. The researcher has overall accountability for the study, but the co-researchers collaborate in selecting the method used and developing a negotiated understanding of a text [31]. Summative content analysis has been used successfully to identify patterns in a document review [34, 35]. In a study on analysis of guideline development manual instructions for implementation, a summative content approach based on an established framework of guideline was validated through review by health professionals and researchers [35]. In this study, the three non-voting members of the FTM-EC extracted quotations on each ICP element from the notifications to researchers while the other two researchers who are/were FTM-EC members also re-examined all proposals to confirm and extract additional text (if any) related to the ICP elements as stated in the notifications and the proposals themselves, and passed them to four coders. The coders included researchers (the two heads of the FTM-EC panels) and co-researchers (two English-speaking personnel working in the ORS, but not in the ethics section). The four coders purposively selected non-repetitive codes and agreed on the pattern of ICP issues raised by the FTM-EC before coding. The consensus on the coding for each of the selected quotations were used as examples of ICP issues in malaria research proposals submitted to and reviewed by the FTM-EC during the previous 5 years.

## Results

### ICP requirements for different study types

Table 1 shows that all IND studies required an ICP, as did about half of the other study types. All study proposals involving minorities and/or children submitted to FTM-EC included an ICP. Half of the studies on other vulnerable populations used archived specimens and/or secondary data, so that no ICP was required. All studies collecting new specimens or new data from questionnaires or interviews required an ICP, whereas studies using unlinked specimens/data did not. Interestingly, only 7% of studies using linked specimen archives and 12% using linked data/records required a new ICP. Most of these types of study submitted authorization for use of specimens/data archived from previous studies.

**Table 1:** Malaria studies reviewed by FTM-EC

Study characteristics	All Protocols Submitted to FTM-EC			
	Reviewed	Approved & Deferred^a^	Required new ICP^b^	
	*N* = 124	*N* = 112	*N* = 63	
	n	n	n	%
Type of Study				
Clinical (IND) trial	14	11	11	100.0
Other clinical study	32	31	17	54.8
Laboratory (basic science) study	49	42	22	52.4
Social/behavioral/epidemiology study	29	28	13	46.4
Specific type of study				
Multicenter study	18	15	14	93.3
International study	6	4	3	75.0
Review together with other IRB	39	34	29	85.3
Involvement of vulnerable population				
Children	21	18	18	100.0
Minority	38	33	33	100.0
Others (pregnant/older/unconscious)	13	12	6	50.0
Data/specimen collection				
Collect new specimens	62	52	52	100.0
Use archived specimens (linked)	28	27	2	7.4
Review medical records/charts (linked)	16	16	2	12.5
Administer questionnaire or interview	9	8	7^c^	87.5
Use specimens/medical records (unlinked)	9	9	0	0.0
Final review outcome				
Approved		106	57	53.8
Deferred		6	6	100.0

Notes:

% shows percentage of deferrals that required a new ICP

^a^Excluded six new protocols pending review; three protocols not approved; three withdrawn by investigator/FTM-EC

^b^Excluded studies using unlinked/left-over specimens/data, or ones where prior consent was obtained

^c^Exempt PIS/ICF for one protocol using Delphi technique (malaria expert consensus)

### ICP elements by study type

Of the 63 studies requiring a new ICP, about 67% raised concerns among FTM-EC committee members about procedures (study activities or specimen/data collection process), about 40% for risk and discomfort, vulnerable status and compensation, and about 15–20% for most other ICP issues (see Table 2). When classified by whether the study used new/linked specimens (54 studies) or linked data/records (nine studies), it was clear that ICP issues, especially study procedures, were raised more frequently among studies using new/linked specimens than among those using linked data/records (77% vs. 33%), even though the sample sizes of the two groups differed considerably. After grouping the studies by whether they involved vulnerable groups, 41 studies had vulnerable populations, and 22 did not. Again, high percentages were found for procedural ICP elements (study activities and specimen/data collection process), risk and discomfort, vulnerable status, and compensation. Studies involving vulnerable populations appeared to have higher percentages with concerns. A comparison of studies that received final approval (57 studies) vs. those deferred (six studies) showed that the main ICP elements resulting in deferral were the proposed study procedures.

**Table 2:** ICP elements raised by FTM-EC by different study types

Concerns about informed consent process	All studies		Use of new or linked specimens				Involvement of vulnerable population				Final review outcome			
			Yes		No		Yes		No		Approved		Deferred	
	*n* = 63		*n* = 54		*n* = 9		*n* = 41		*n* = 22		*n* = 57		*n* = 6	
	n	%	n	%	n	%	n	%	n	%	n	%	n	%
Purpose of the study	10	15.9	8	14.8	2	22.2	8	19.5	2	9.1	9	15.8	1	16.7
Duration of the study	2	3.2	2	3.7	0	0.0	0	0	2	9.1	2	3.5	0	0.0
Procedure for study activities	42	66.7	39	72.2	3	33.3	30	73.2	12	54.6	36	63.2	6	100.0
Potential risk/discomfort	26	41.3	22	40.7	4	44.4	19	46.3	7	31.8	23	40.6	3	50.0
Method for minimizing risk	4	6.4	3	5.6	1	11.1	3	7.3	1	4.6	4	7.0	0	0.0
Potential benefit	7	11.1	6	11.1	1	11.1	6	14.6	1	4.6	6	10.5	1	16.7
Concern about vulnerable status	25	39.7	23	42.6	2	22.2	23	56.1	2	9.1	22	38.6	3	50.0
Voluntary participation	13	20.6	11	20.4	2	22.2	7	17.1	6	27.3	13	22.8	0	0.0
Right to withdraw from study	13	20.6	10	18.5	3	33.3	8	19.5	5	22.7	12	21.1	1	16.7
Concern about confidentiality	13	20.6	12	22.2	1	11.1	9	22	4	18.2	13	22.8	0	0.0
Coverage of cost (if any)	10	15.7	10	18.5	0	0.0	8	19.5	2	9.1	9	15.8	1	16.7
Justification for compensation	26	41.3	25	46.3	1	11.1	21	51.2	5	22.7	22	38.6	4	66.7
Contact name/telephone for emergency	16	25.4	14	25.9	2	22.2	13	31.7	3	13.6	13	22.8	3	50.0
Notifiable body for protocol non-compliance	4	6.5	3	5.7	1	11.1	4	9.8	0	0	4	7.1	0	0.0

Note: Percentages were calculated for each type of study

Examining the ICP elements raised by the FTM-EC when they read the proposals and the other documents written for study participants, Table 3 shows that clinical trials testing new drugs (IND) had higher percentages than other types of study for critical informed consent. About 90% of IND studies did not explain the study procedures/activities clearly in layman terms. Interestingly, about half of the IND proposals had issues related to incomplete information on potential risks/discomforts but no IND studies attracted comments on potential benefits, which were usually clearly stated. About 45% of the IND studies had concerns on ICP issues related to confidentiality and the vulnerability status of the study participants. Similar critical ICP issues were observed, but with a lower percentage, among biomedical/non-IND clinical proposals that raised such issues. Regarding laboratory or basic science studies, as the researchers had to collect data/specimens from study participants, the ICP issues still persisted on incomplete or unclear information related to study procedures, mostly on the methods of obtaining data/specimens. ICP related to the involvement of vulnerable study participants appeared in 45% of basic science proposals. About 30% of such studies raised concerns regarding voluntary participation and cost coverage required for study participation. Among malaria studies with social- and behavioral-science approaches, high percentages were shown for ICP elements regarding study activities, potential risks/discomforts, vulnerable status and voluntary participation, respectively. The issue related to the amount and timing of compensation provision was also raised in all types of study.

**Table 3:** ICP elements raised by FTM-EC by different study types

Concerns about informed consent process	Clinical (IND) study		Other clinical study		Laboratory (Basic Science) study		Social/Behavioral/Epidemiology study	
	*N* = 11		*N* = 17		*N* = 22		*N* = 13	
	n	%	n	%	N	%	n	%
Purpose of the study	1	9.1	1	5.9	4	18.2	4	30.8
Duration of the study	0	0.0	2	11.8	0	0.0	0	0.0
Procedure for study activities	10	90.9	12	70.6	12	54.5	8	61.6
Potential risk/discomfort	6	54.6	7	41.2	7	31.8	6	46.2
Method for minimizing risk	2	18.2	0	0.0	0	0.0	2	15.4
Potential benefit	0	0.0	0	0.0	4	18.2	3	23.1
Concern about vulnerable status	5	45.5	5	29.4	10	45.5	5	38.5
Voluntary participation	1	9.1	2	11.8	6	27.3	4	30.8
Right to withdraw from study	3	27.3	1	5.9	6	27.3	3	23.1
Concern about confidentiality	5	45.5	2	11.8	4	18.2	2	15.4
Coverage of cost (if any)	1	9.1	3	17.7	6	27.3	0	0.0
Justification for compensation	7	63.6	5	29.4	8	36.4	6	46.2
Contact name/telephone for emergency	4	36.4	7	41.2	3	13.6	2	15.4
Notifiable body for protocol non-compliance	1	9.1	1	6.3	1	4.6	1	7.7

### Content analysis of ICP elements

The authors and the coders agreed on a classification system for the pattern of ICP issues discussed during board meetings and raised by the REC according to the types of REC requests for answers or responses from the researcher who submitted the proposal. In an attempt to group ICP elements from international standards [2, 6–8, 11], the authors classified them into 5 main categories: (a) purposes and procedures of the study, (b) risk and benefits, (c) vulnerable populations, the voluntary nature of research, and withdrawal, (d) confidentiality and contact, and (e) cost and compensation. The purposes and procedures cover how well the researchers describe or communicate with study participants in terms of study objectives and research methodology. Explanation of all possible risks/discomforts and declaring potential or no direct benefits are core beneficent information that should be clearly conveyed to study participants. Considerations of the vulnerable nature of the study participants and asking for voluntary participation were both required ICP issues reflecting how the researchers respected the study participants. Confidentiality and contact issues reflect the protection of study participants. Cost covers issues related to the anticipated expenses/payment that the study participants had to pay on their own, while compensation can be monetary and non-monetary means provided for study participants’ contributions/efforts, for study-related injuries, and/or for the loss of their own time/earnings. The quotations related to the five ICP elements in Tables 4, 5, 6, 7 and 8 were extracted from the original proposals and/or notifications to researchers and then given to the study coders to assess the nature of the ICP issues.

**Table 4:** Examples of ICP elements – purposes and procedures

ICP Issues Raised by the REC	Type of request			
	C	E	R	P
Purpose				
Stating the overall aim in the Participant Information Sheet as “to make pregnancy safer” may be too ambitious, because this study rarely involves pregnancy.	X		X	
Please state the objectives of the study clearly in the Participant Information Sheet, ensuring they are the same as the proposal, but using simpler and more easily understandable wording.	X		X	X
In the Participant Information Sheet objectives, it is stated: “We are trying to find the lowest dose at which we can stop you from passing malaria to mosquitoes.” Please revise this sentence as it may confuse some participants.	X		X	X
Rearrange the Participant Information Sheet according to the various aspects of the research activities. Please explain each part clearly, such as what will be done in the genetic study, molecular marker testing, and PK study.	X	X	X	
Add check boxes in the Informed Consent Form to indicate in which part of the study the participants would consent to participate, since there are several objectives for the study, including participating in resistant parasite clearance study, participating in PK study, participating in genetic determination/molecular marker, consent for home visit and data collection for GIS. Also provide a separate check box when asking for permission to keep leftover blood (if any) for future study.	X		X	
In the Informed Consent Form, there should be a statement that the study participant has been informed of and understood the study objectives and procedures thoroughly, and it should also mention that the participant has the opportunity to ask any questions and get satisfactory answers.	X		X	
Procedure – intervention				
In the treatment section, specify clearly how the dose escalation will be performed; the researcher should also mention that some of the patients may receive less than or more than 75 mg of the drug.	X		X	
The study is rather complex, mixing different groups of study participants and different types of specimen collection. Please explain separately what will be done: for patients – the study participant recruitment and the informed consent process that will be conducted; for the general population in the community – informing the community leader and recruitment process; and for the hospital director – permission to use left-over specimen(s).	X	X	X	
In the research proposal, it was mentioned that the patients will be monitored using the Holter Monitoring tool; this should also be mentioned in the Participant Information Sheet.	X		X	
In the procedure section about “What will happen to your child”, state the specific frequency of visits instead of “regularly”. Also describe the procedures to be undertaken at each visit.	X	X	X	
It was stated in the risk section that, “before receiving primaquine, the research team will always test your blood”. However, primaquine will be administered after Day 3 or Day 5. How does the research team plan to handle patients who are not admitted to the study site? Please describe clearly the procedure for follow-up and the performance of hemolysis in such cases.	X	X	X	
Specify the risks for all medications used in the study and state them clearly in both the research proposal and the Participant Information Sheet.	X	X	X	
Specify clearly how to measure the treatment dose, who will measure it, and where the treatment is monitored.	X	X	X	
Procedure – recruitment				
How will volunteers for the PK study be selected?	X		X	
Please detail the process by which immigrants will be invited to participate in the research study.	X	X	X	
The method for approaching participants is inappropriate. The Principal Investigator should invite patients presenting at the clinic [to be involved] and ask for their permission before accessing their electronic clinical records. Is there a translator responsible for meeting the participants and explaining the study, since all of the research team is foreign? Please explain.	X	X	X	
In the informed consent process, describe clearly who will explain and answer queries about the study procedures. The patients have to understand fully that “the research procedures are different from routine treatment and care services”.	X	X	X	
The Participant Information Sheet for healthy volunteers and for those with acute febrile illness should be separate, as the content and procedures involved are different.	X		X	
The Participant Information Sheet states that the total participants will comprise 30 adult males. This conflicts with the inclusion/exclusion criteria in the proposal.	X		X	
Check and confirm the number of participants to be recruited. In the Participant Information Sheet, the number is 500; however, in the methodology section of the research proposal, it is 70–100.	X		X	
Informed Consent Form Page 2/3, Remark 2: stated that “The person explaining or reading the statement must not be a doctor”. The words “a doctor” should be “the doctor administering treatment”.	X		X	
Procedure - data/specimen collection				
Since blood will be drawn 24 times consecutively (total = 140 ml), the researcher should clearly inform the study participants of the relevant procedures.	X		X	
How much blood will the research team collect from the participants? How many times? The figures are conflicting both in the protocol and the Participant Information Sheet. Please make it clear and consistent. Specify how many participants will give blood for this study.	X	X	X	
Why should blood be drawn 13 times (in weeks 1, 2, 4, 6, 8, 10, 12, 14, 16, 18, 20, 22 and 24) – especially for young children? If there is no re-infection, the antibody will not change much; please reconsider a blood draw of no more than once every 1 or 2 months. Re-infection can be detected from other clinical symptoms.	X		X	
Regarding the follow-up outcome assessment for any study volunteer who falls pregnant during the trial period, please state explicitly for how long the pregnant volunteer is withdrawn from taking the testing drug in the study.	X	X	X	
Specify clearly that the research team will interview only the head of the household.	X		X	
The researcher should tell the study participants that their medical records will be examined and they will be interviewed using a structured questionnaire, and that the healthcare provider will visit their house to collect household information.	X	X	X	
How does the researcher intend to collect leftover patient blood from hospitals under the Thai Ministry of Public Health? The Director of each participating hospital must provide permission in writing.	X		X	
Why is a genetic sample collected at Visit 1 (screening visit)? This type of sample should be collected at Visits 2–7 (enrollment and follow-up visits), because some volunteers may not be selected for participation in the study. Otherwise, a specimen from an individual who does not pass the screening test will not be used in the study at all. This procedure should be clearly explained in the Participant Information Sheet and Informed Consent Form. There should also be a separate Participant Information Sheet/Informed Consent Form or separate check box for this (genetic sample) study objective.	X	X	X	

Note: *C* clarification, *E* elaboration, *R* revision, *P* paraphrasing

**Table 5:** Examples of ICP elements – risks and benefits

ICP issues raised by the REC	Type of request			
	C	E	R	P
Risk and discomfort				
Please avoid using the statement “Do not worry about the amount of blood taken, because it is very small” (Page 4/6). In fact, 2 mL will be taken per visit, which is not a very small amount. Also, state the blood volume in teaspoon(s)/tablespoon(s) in the summary table for blood collection.	X	X	X	
In the risk section of the Participant Information Sheet, please amend the text to read: “Some questions may make you feel uneasy or uncomfortable. You may refuse to answer any question at any time.”	X		X	
In the risk section of the Participant Information Sheet, the researcher should state that chloroquine (CQ) alone is not a standard treatment, and that if the patient is randomized to this group, the risk of relapse may increase.	X	X	X	
In the risk & discomfort section of the Participant Information Sheet, the researcher should mention that, in addition to bruising, there may be a risk of becoming infected, and if infection occurs, how the research team plans to handle it.				
Please state clearly that there may be a risk of a hemolysis event for any volunteer with glucose-6-phosphate dehydrogenase deficiency (G6PD deficiency), and how such an event would be identified; this is important for enabling potential study volunteers to make an informed decision on participating in the study.	X	X	X	
The researcher should reword the phrase “thus there is no risk” to “the risk of mosquito bite is minimal; however, if you are bitten by a mosquito and are infected with malaria, the researcher will….” Explain what the researcher will do and who will be responsible for the costs.	X	X	X	
On Page 2 of 10 of the Participant Information Sheet (English version), it is stated that “We still want to check to see that there are no unexpected side-effects from XXX treatment”; this statement is not quite correct. The purpose of the study is to test dose deceleration; therefore, patients should be informed of any associated risk. This should include, for example, that treatment XXX is not the current standard treatment and that patients may run the risk of not being cured or deteriorating.	X	X	X	
Please add a statement that research participants may suffer hemolysis or anemia, and may receive a placebo.		X	X	
In the statement addressing potential risk and discomfort, “If there is any side effect ... the researcher will provide treatment and care immediately”, please change to “… should a side effect occur, the researcher will be responsible for treatment and care. You will not have to pay for any cost related to the event”.	X	X	X	
Please specify the risk(s) of all medications used in the study, and present them clearly in both the research proposal and the Participant Information Sheet.	X	X	X	
In the risk and benefit section, it should not be stated that “All study procedures are routine and pose no additional risk to the study participant”, because these extra investigations are not routine procedures.	X		X	
Minimizing risk				
In the description of the study procedure: is venipuncture necessary, since after admission the researcher will only need a specimen for blood film and filter paper, which could be obtained from a finger prick?	X			
The Participant Information Sheet should include details of what the patient should do if he/she gets re-infected after discharge from hospital.	X	X		
Please specify what will be done should there be a serious adverse event.	X	X		
In the event that disease is detected, specify the list of diseases for which the PI will provide free treatment/intervention. Also include the management of the intervention and list the diseases for which the PI will transfer the patient to a suitable hospital.	X	X	X	
Please revise the statement “No risks or discomforts are anticipated from taking part in the study”. Participants will require a long time to answer the questionnaire used in this study. Please inform the study participants about time requirements and their rights in voluntarily answering any sensitive questions in the questionnaire. Particularly for the focus group and in-depth interview, please mention that participants have the right to withdraw or not answer any questions.	X	X	X	
In the Participant Information Sheet, it is stated that about 10 drops (500 μl) of blood would be collected for the current study, with blood for future use. Does that mean the researcher plans to draw more blood than is actually needed for the current study? In addition, for volunteers aged >13 years, why does the researcher need another 5 ml, when the 10 drops (500 μl) should be enough. Please reconsider the amount of blood required for this study.	X	X	X	
The risk-benefits of the study outlined in the protocol should include clear information on the provision of treatment/intervention/medical care to children when abnormalities are detected.	X	X		
Please explain co-morbidity and potential side effects, since the local people may not understand what the researcher is talking about.	X	X		
Page 11/24: Treatment for recurrent *Plasmodium vivax* (PV) infection will be the same as open-label study drug(s). If infections recur, it might suggest that the drugs taken previously were not appropriate; therefore, a standard drug regimen should be used instead to treat a recurrent episode. Why do you not want to use the standard regimen to treat patients with recurrent PV?	X		X	
If participants show symptom(s) of clinical hemolysis, please specify clearly how you will treat the symptom(s).	X	X	X	
Please specify the alternative (standard) treatment, mentioned on page 1 of the Informed Consent Form.	X	X	X	
Benefits				
Please add a benefit section to the Participant Information Sheet, describing the potential benefits for the study volunteers and/or others, such as “… you or your child might not receive any direct benefit from participating in this study, but the results of the study could be used for …”	X	X	X	
What are the potential benefits of this study to others? Please describe.		X	X	
A “small sum of money” is not a benefit to the patient; it is rather compensation and a token of appreciation. Please delete from the benefit section.			X	
In the description of direct benefit, please change the sentence, “You will receive second-line treatment” to “You will be referred/sent to a malaria clinic or hospital for treatment.”	X		X	
In the benefit section of the Participant Information Sheet, the researcher should delete “You will not get vivax malaria again unless you get infected from a mosquito bite”. Based on the content flow in this part, the sentence might better be “You will still receive primaquine, even though you are not participating in this study”	X		X	

Note: *C* clarification, *E* elaboration, *R* revision, *P* paraphrasing

**Table 6:** Examples of ICP elements – vulnerable populations, the voluntary nature of research, and withdrawal

ICP issues raised by the REC	Type of request			
	C	E	R	P
Vulnerability				
The language in the Participant Information Sheet and Informed Consent Form/Informed Assent Form should be revised to make it appropriate to age and educational level.	X		X	X
There should be separate forms for adults (aged >18 years) and persons aged 15–18 years.			X	
Please provide local-language Participant Information Sheets, Informed Consent Forms and Informed Assent Forms (Karen, Burmese, and Thai versions), because potential participants must have a very clear understanding before signing informed consent. Please also provide certified translations of these documents.	X	X	X	X
Please elaborate further on the recruitment process. How many study participants in each village can speak and understand Thai? Please specify who will explain or translate the study to the participants. Please also provide a local-language Patient Information Sheet.	X	X	X	X
With regard to the objective of the study, does this study need a sample size of 100, or is it too many? If the researchers want to draw blood simply to determine long-term culture, there is no need to conduct the study with children, and no need to draw >5 ml of blood.	X		X	
Is it necessary to perform venous blood collection with children aged >5 years? Can the PI increase the age of the participants and collect blood samples by finger prick? Please also consider decreasing the amount of blood for RT-PCR and blood smear tests to <2 ml, since each test does not require much blood.	X		X	
Drawing 5–10 drops of blood from the index finger of a small child may cause bruising, and may risk infection with a deeper puncture. Normal medical practice is to draw blood from the heel of a small child aged 6 months to pre-walking age.	X		X	
Voluntary participation				
In the Informed Consent Form for children aged 13–18 years, please add “I fully understand that I can refuse to participate in this study; nobody is forcing me to enroll in this study, not even after my parents/guardians provide consent for me to participate”.	X	X	X	X
The voluntary participation section (Informed Consent Form page 2) currently states “if you decide not to participate in this study, there will be no impact on any treatment that you will get…”. Please change this to “if you decide not to participate in this study, you have the right to do so…”, because this study is conducted among healthy students in school and no treatment is involved.	X		X	X
The statement about study participation could start with “Participation in this study is voluntary. You can make your own decision freely. You can refuse to participate in this study. If you do so, you will not ….”	X	X	X	
Please consider changing the sentence “Your spouse has to give informed consent.” This sentence may be inferred to mean that the spouse is obliged (under some duress) to sign the Informed Consent Form.	X		X	
The researcher should inform the study participants that they have to right not to answer questions they feel are inappropriate.	X		X	
Withdrawal				
In the “withdrawal” section, if the participant asks to withdraw in the middle of the study, after specimen collection, how does the researcher plan to handle such specimens? Please detail clearly.	X	X	X	
In the 2nd sentence of the “withdrawal” section, “You have the right to withdraw any time without any penalty”, the words “without any penalty” are inappropriate and should be changed to “… and you will still receive standard treatment and care. Not participating will have no impact on your existing right to receive treatment and care.”	X		X	
The researcher should indicate that the owner of the specimen has the right to withdraw his/her specimen from the specimen bank.	X		X	
Please specify clearly: when patients withdraw from the trial, will the information in their medical records be used?	X		X	

Note: *C* clarification, *E* elaboration, *R* revision, *P* paraphrasing

**Table 7:** Examples of ICP elements – confidentiality and contact

ICP issues raised by the REC	Type of request			
	C	E	R	P
Confidentiality				
In the confidentiality section, the researcher should consider starting the section with “Your personal information will be kept confidential by ….”	X		X	
Please specify in the Participant Information Sheet and Informed Consent Form who will be authorized to have direct access to medical records.	X		X	
The Participant Information Sheet currently states that only persons in the pharmaceutical company (international) will have authorized access to pharmaco-genetics; this should be reconsidered, to include the IRB and other stakeholders at national level.	X		X	
The Informed Consent Form states “I give permission for authorized persons to access my medical records, such as the study team, representatives of the research sponsor, the Ethics Committee, and monitors.” Please specify precisely who are actually involved and their roles and responsibilities, after the words “such as.”	X		X	
The Informed Consent Form and Case Report Form may not include personal addresses.	X		X	
Is there permission to use data in the patient’s medical records? How does the researcher plan to handle the confidentiality of patient information?	X	X	X	
This study is a one-time cross-sectional survey with one blood draw; since there is no follow-up visit, why does the researcher want to collect data on study volunteers’ addresses (even though it is stated in the proposal that such data will be kept in a secure facility with limited access)?	X		X	
There should be no link from an individual to a specimen in this study. Each specimen should be coded with a code number. The proposal should state clearly who has access and who may retrieve data for the researcher, and what coding procedure will be used.	X		X	
Please explain the statement “All samples were kept in a bag without a clear label in each one”. What does this mean?	X		X	X
Please specify for how long the data will be kept, and when they will be destroyed.	X		X	
If the plan is to keep leftover blood in additional locations, please specify where in both the research proposal and the Participant Information Sheet.	X		X	
Contact information				
In the Participant Information Sheet, there is no address for contacting the study doctor. Please add one of the investigating team as the contact person.	X		X	
Emergency contact information should be provided to all participants, with a mobile phone number. The phone number of the local physician should be provided.	X		X	
The contact person stated in the proposal is Dr. XXX, but in the Informed Consent Form and Assent Form it is Dr. YYY. Please make it clear. Do both of them speak Karen, Burmese and Thai? Is there a translator? Please specify.	X		X	
The contact person in the Participant Information Sheet is Dr. XXX, but her name is in the list of study investigators.	X		X	

Note: *C* clarification, *E* elaboration, *R* revision, *P* paraphrasing

**Table 8:** Examples of ICP elements – cost and compensation

ICP Issues Raised by the REC	Type of request			
	C	E	R	P
Cost				
In the “cost” section, it is currently stated that “The pharmaceutical company will pay for appropriate treatment.” Who will make the decision regarding the appropriateness of the condition or the treatment for payment? Please explain.	X	X	X	
In the “cost” section, in relation to the costs the participant must pay, the researcher should add, “should there be an adverse incident related to the study procedure, the research team will be responsible for it.”	X		X	
It is currently stated, “The cost of treatment and laboratory according to the prescription of the hospital physician will be paid by yourself.” This should be more precisely stated “…., if such treatment and care are not related to the study.”	X		X	
Please state that the investigator will pay for treatment if the study participant shows no response to the study drug.	X		X	
Research-related injuries should cover the costs of potential life-long or long-term care for study-related injuries.	X		X	
Compensation				
Please reconsider whether compensation is fair for participants staying at the hospital and participants attending a follow-up visit (100 Baht (3 US $) for all).	X		X	
Please reconsider the compensation provided to research participants; 150 baht per visit and 300 baht for an overnight stay is not likely to be appropriate.	X		X	
It is not clear whether compensation of 200 Baht for travel costs will be paid at the end of the study or per visit. Patients will have to return for follow-up twice.	X		X	
In the compensation section, please specify the amount(s) of money that will be provided to compensate participants.	X		X	
The rationale for compensation, which is stated as “for participation in this study” is inappropriate, because it appears to be an inducement. Please reconsider. Suggestion: it might better be “for time and travel”.	X		X	
In section C7 (compensation), it is currently stated that “Volunteers will receive a gift package… snack and insect repellent.” Please consider adding that this gift is a token of appreciation for participating in the study rather than compensation.	X	X	X	
Compensation provided to research participants: 100 Baht per visit is probably inappropriate, because participants may have to be off work the whole day. Please consider an amount of 300 Baht, which reflects the daily minimum wage.	X		X	
Why is the compensation for acute febrile patients 200 Baht, while healthy controls get 300 Baht? Please clarify.	X		X	

Note: *C* clarification, *E* elaboration, *R* revision, *P* paraphrasing

Given that the ICP is mainly about effective communication between the researcher and study participant, REC directions to ensure adequate and comprehensible information exchange with study participants were classified into four types of request: (1) clarification, (2) elaboration, (3) revision, and (4) paraphrasing. A notification for clarification requests an explanation of unclear, conflicting, or contradictory information; if such an issue is raised by the REC, approval remains pending, with neither acceptance nor non-acceptance. A REC request for elaboration requires the researcher to provide more information about the issue raised. The issue remains pending and probably acceptable with additional information. Revision is requested where the REC has a major, unacceptable concern and requests a change in the content or methods proposed. A notification for paraphrasing requests was issued when the researchers needed to rephrase text (content) about which the REC had some concerns. This includes issues where the current wording is inappropriate because of implied coercion, or potentially misleading in that it creates a false impression, or overstates or exaggerates the facts. Examples of quotations related to each ICP element from the notifications to researchers are shown in Tables 4, 5, 6, 7 and 8. The four coders reached consensus on the type of request for each quote.

#### Purposes and procedures

Issues related to the purpose of the study in the ICP were generally about the stated purpose in the objective section of the proposal. Researchers usually tried to simplify the study objectives for study participants when writing the PIS and ICF/IAF. At times, the messages became distorted, unclear, or even misleading. Problems about procedures in the protocol, PIS, ICF/IAF, or other ICP documents/materials were usually about the intervention or treatment to be used in the study, the recruitment process for study participants, and the specimen/data collection methods. FTM-EC typically requested clarification, elaboration, or revision of these ICP elements (see Table 4).

#### Risks and benefits

Most of the risk-related ICP issues discussed and raised by FTM-EC concerned the participants’ understanding of the risks and/or the lack of adequate information about the risks of side effects and adverse events. Many proposals did not mention the rescue method and treatment or care that would be provided if an unexpected adverse event occurred. They also did not discuss alternative methods that might be available. Several concerns related to unnecessary and/or overly invasive procedures for certain groups of study participants. The benefit of the study could be provided directly or indirectly to the study participants; many proposals which were basic science and epidemiology studies, omitted or were confused about this ICP element. Several proposals confused study benefit with compensation for study participants. The FTM-EC typically requested clarification, elaboration, or revision of these ICP elements (see Table 5).

#### Vulnerable populations, the voluntary nature of research, and withdrawal

Some proposals were somewhat insensitive to the needs of vulnerable populations. While reviewing the PIS, ICF/IAF and other documents in English and/or Thai, FTM-EC always requested such documents in ethnic languages if the study was to be conducted among ethnic groups in border areas. Such documents might be submitted after the formal ethics committee review sessions, and the research office ensured their certified translations. Almost all proposals mentioned voluntary participation, but some needed to be paraphrased. Many proposals, although they involved clinical investigations, did not mention the right to withdraw and the withdrawal-replacement process. Some studies asked to archive leftover specimens for future use, but did not mention that the study participants could later decide to withdraw consent. The FTM-EC usually requested clarification and/or revision in such cases (see Table 6).

#### Confidentiality and contact

Researchers appeared to be well aware that study participants need to be informed about confidentiality provision. Almost all proposals contained such confidentiality information, so that the FTM-EC seldom needed to raise the issue of confidentiality in proposals. This ICP element appears to be well understood among researchers. Proposals to use existing linked data often generated concerns about who could access and/or extract data for use (i.e., the relevant or authorized person). Proposals requesting the archiving of specimens/data for future use also raised confidentiality issues. A few proposals had problems with furnishing contact details for untoward or unethical incidents. FTM-EC generally requested clarification and revision in such matters (see Table 7).

#### Cost and compensation

Most proposals did not require self-payment by study participants. The few studies that required payment did not state this clearly. Compensation appeared to be one of the main ICP elements raised by FTM-EC. Most of the time, the amount provided was not commensurate with the procedures and time loss in the study. In several proposals, the amount, procedures and timing planned for providing compensation to study participants at each visit and/or throughout the study period were unclear. FTM-EC usually requested the researchers consider revisions in such cases (see Table 8).

## Discussion

The ethical foundation of any ICP is respect for the individual, recognizing that every competent individual is entitled to make their own decision freely based on an adequate understanding of what the research and their participation involves [2]. A study of ICP awareness analyzed the behavior of 68 patients during the informed consent process of a clinical trial. The findings revealed that about one-third of the patients did not ask any questions [36]. Although these findings are specific to one study, they may reflect some common issues: lack of interest, lack of awareness, and lack of understanding of the ICP among study participants. Ethical guidelines for clinical research [2, 3, 6–8, 11] oblige RECs to ensure that the relevant information about the research is thoroughly explained in an understandable way to study participants. This study shows that all clinical (IND) studies, and epidemiological studies collecting new information from the study participants, attracted REC comments on elements of the ICP. A study on comments raised during ethical review among protocols of drug trials also reported that about 70% of the protocols received comments about participant information, consent forms, and supporting documentation [21].

Although the collection and storage of specimens for study, and the future use of archival or leftover specimens, are typically thought to involve minimal risk in malaria research, a considerable amount of information must be conveyed during the ICP. The use of archived specimens/data, particularly where genetic information is involved, is becoming more common in malaria research. These types of study still present a challenge to traditional ideas of informed consent, because several alternative models for ethical consideration exist [29]. The literature suggests that RECs in some jurisdictions can determine whether sharing a participant’s data for research purposes is consistent with the original consent or not [30]. At FTM-EC, if the archived specimens/data were unlinked or consent had previously been given for future use, and was authorized by the specimen/data owners, then the committee had no further concerns about ICP elements. In most cases, where a proposal sought to retain leftover specimens or data for future use, the FTM-EC asked the researcher to explain the procedure clearly in the PIS/ICF/IAF, and provide a separate check-box to enable participants to specify whether they would permit the retention of their specimens/data for future use.

The Helsinki Declaration states “*Medical research involving human subjects must conform to generally accepted scientific principles, be based on a thorough knowledge of the scientific literature, other relevant sources of information, and adequate laboratory and, as appropriate, animal experimentation”* [1]. This study found that FTM-EC had most ICP concerns about the procedures or study activities. Particularly in clinical trials, an informed decision to participate will require study participants to be thoroughly informed about what will be done to them (procedures), and how the research plan will work [6]. As well as a description of the intervention (if any) to be tested, and the procedures to be followed, information for research participants should include an explanation of the purposes of the research, the expected duration of the subject’s participation, and the approximate number of research participants to be enrolled [6, 7, 26, 37]. The ICP should also state clearly that the study involves research that is not simply routine treatment and care. For many proposals, the difficulty for the researchers and REC review is how to make the scientific procedures comprehensible to the general population or to non-scientists [22]. The guidelines suggest that the REC should ensure that technical and scientific terms are adequately explained or common terms substituted, and that the ICP should be translated from complex scientific concepts into simple terms [2, 6, 11, 22]. This problem was frequently raised by FTM-EC about malaria research proposals. The guidelines suggest constructing consent materials for low health literacy by ensuring the reading level for documents does not exceed eighth-grade level (approximately 12–13 years old) [6]. The main concerns were word choice, pronoun use, and terminology. The literature recommends that ICP documents be written in a conversational style that mirrors verbal communication between investigator and participant [37]. Since malaria research is frequently conducted among different ethnic groups living in areas where the disease is endemic, FTM-EC raised several relevant ICP issues, e.g., that a translation of the study procedures be clear and simple but still cover all of the important activities. The literature also suggests that researchers and RECs should take into consideration the impact of having diverse study participants [38].

One of the most important principles for research with human subjects is that all such research “*must be preceded by careful assessment of predictable risks and burdens to the individuals and groups involved in the research in comparison with foreseeable benefits to them and to other individuals or groups affected by the condition under investigation”* [1]. It is an ethical requirement, to minimize the possibility of exploitation, that study participants should not receive unfair levels of benefits or an unfair burden of risks/discomforts. Before making the decision to participate, potential study participants should be fully informed of any risks and/or benefits they may or may not directly/indirectly receive while participating in the study. Concern about the balance of risks and benefits does not focus exclusively on health. The guidelines also suggest that the consequences may involve pain, discomfort or fear, and affect employment, social, or personal life [16, 39]. Reports have suggested that even after signing a consent form, patients frequently did not understand the risks, benefits, and alternatives involved in the procedures for their course of treatment [13, 40–42]. In a retrospective review and analysis of negligence claims against doctors, the primary allegation in 71% of cases was that the clinician failed to mention or properly explain the risks of complications [43]. Many studies have revealed a poor level of understanding among study participants, such that they were not even aware that they were participating in a research study and/or believed that the research was conducted primarily for their own benefit rather than for generalizable knowledge or the benefit of others [44]. The literature suggests that it is important to maintain a balance between risk and benefit in the information provided, and to avoid any misconceptions. Although the research may benefit society in the long term, the interests of the individual always prevail over those of science and society [15, 16]. The results of the content analysis for this study suggest that the risks and benefits were sometimes not clearly or thoroughly explained to study participants, and the FTM-EC therefore requested elaboration and revision of these elements.

Many of the malaria research studies reviewed by FTM-EC involved vulnerable populations (particularly ethnic minority groups, children, and pregnant women) since the disease is prevalent among populations in the country’s border areas with Myanmar and Cambodia. Other ethical guidelines listing vulnerable populations include those with poor mainstream language skills, low levels of literacy, some form of disability or cognitive impairment, or culturally and linguistically diverse backgrounds [1–3, 8, 9]. The WHO guidelines suggest that reasonable efforts should be made to ensure that the needs of vulnerable populations are adequately addressed. These needs include difficulty accessing services and resources, need for alternative communication strategies, impact of stigmatization and discrimination, disproportionate burden of epidemic response measures, and disproportionate need for limited resources [45]. One of the common rules for safeguarding the rights and welfare of these vulnerable participants is to scrutinize the potential for undue influence or coercion [46, 47]. The participation of those who lack capacity or are unable to make independent, free decisions needs to be agreed by someone who is independent of the study and can properly assess the potential participant’s interests [6, 10, 11, 28, 48]. Members of ethnic minority groups and/or children residing in areas where malaria is endemic and low-resource settings may constitute a more complex population, sharing some but not all of these characteristics. FTM-EC requested that several researchers should reconsider or revise ICP documents, and asked for certified translations of the documentation into other languages for particular populations. A guideline [6] suggests that the informed consent document should be in a language that the subject or their authorized representative understands. A translator or interpreter can facilitate conversations with study participants, but routine ad hoc translation of the consent document should not be substituted for a written translation [6]. Another suggestion in the literature, which might be useful for dealing with this population, is a technique to enhance effective communication (the “teach back” approach), where the researcher checks understanding by asking *“Can you tell me in your own words?”* [49].

From an ethical perspective, it is essential that participants are recruited voluntarily into research studies. In many cultures, especially where the patient–physician relationship is dominated by the physician’s authority, this may be questionable [36]. Participant consent is legally valid and professionally acceptable only if they have agreed to participate without pressure or coercion, and participants’ right to decline to take part or to withdraw at any time without reprisal should also be respected [1, 50]. Most proposals submitted to the FTM-EC did include a statement addressing this, but some used inappropriate wording that required paraphrasing. For example, in a study monitoring, clinically and genetically, fetuses and babies of malaria-infected pregnant women with long-term follow-ups before/after delivery, it was stated that their spouse would “have to” give informed consent. When a study was conducted among a cross-border minority population in the only-one-choice basic healthcare facility located in a remote and low-resource setting, such wording might coerce both pregnant women and their spouse to participate, even though the wording derives from the research physicians/nurses, not the obstetrician in charge at the facility. The wording could be paraphrased such that women should take some time for discussion and make the decision together with their spouse whether to participate in the study.

The elements of the ICP relating to privacy and confidentiality must be assessed by the REC. This is key to respect for personal autonomy, which has been defined more widely than liberty (which is freedom from obligation, absence of external causation, and independence), to include dignity, integrity, and individuality [51]. The Declaration of Helsinki states *“Every precaution must be taken to protect the privacy of research subjects and the confidentiality of their personal information”* [1]. In the guidelines for Good Clinical Practice [11, 50], if records can identify the subject, they must be kept confidential and if the results of the trial are published, the individual’s identity must remain confidential. This study found that many proposals using secondary data prompted requests for the clarification of confidentiality issues. One guideline on the use of secondary data suggests that it would be sufficient and practicable to disclose only anonymized or coded information. When identifiable or linked information was desired, however, a supporting rationale would be needed [50]. The committee’s request for clarification or elaboration typically covered the nature of the information to be disclosed, what use will be made of the information, how many people will have access to the information, and the confidentiality and security arrangements in place [50]. A few proposals neglected to include contact details (i.e., address and telephone number of the principal investigator and the ethics committee reviewing the proposal), for use if study participants were not treated as described in the ICP documents. Guidelines for good clinical practice [11] make clear that an explanation is required of who to contact for answers to pertinent questions about the research and participants’ rights, or should there be research-related injury.

This study found that about half of all proposals were asked to reconsider participant compensation. In almost all cases, the compensation was too low. This may be because the researchers thought that the study procedures were not particularly invasive or time-consuming, and that the costs of living for participants in remote areas were low. While there is no definitively right or wrong answer on this, researchers should plan carefully and consider the interests of study participants. The Declaration of Helsinki [1] states, *“Appropriate compensation and treatment for subjects who are harmed as a result of participating in research must be ensured”*. Other guidelines have suggested that research involving more than minimal risk should explain whether compensation and medical treatment would be made available in the event of any injury [2, 11, 44]. WHO guidelines state that it is ethically acceptable and appropriate to reimburse study participants for any costs associated with participation, including transportation, child care, or lost wages and time [2]. The recommendation, however, states that the amount should not constitute an inducement to prospective participants to consent. If compensation is not provided, the rationale for this should be explained to study participants.

The results of this study were based on an analysis of the information or content in the proposals. The ICP is, however, more important once the study has actually started. The literature contains suggestions about how to monitor and improve the process of informed consent, which is a responsibility of all stakeholders involved in conducting the research, including investigators, research institutions, sponsors, and ethics committees. Before obtaining consent, the investigator should assess whether the study participants have been given all the necessary information, and that they understand the details and implications of what is proposed [39]. An investigator should only obtain consent from a potential research subject if they have been given enough time to make a decision [6]. When conducting research involving people from diverse backgrounds, the process of obtaining consent should be culturally appropriate and sensitive to community concerns. This can be done, for example, by involving the public in checking the language used [51]. At the institutional level, the ICP can be improved by providing a formal training program on effective communication, simplifying the content, length and language of informed consent documents, and implementing policies for vulnerable populations [13]. It should be emphasized that informed consent is an ongoing process. The researchers must ensure that participants continue to understand the research in which they are involved by providing new or additional information that might affect their decision to continue to participate [23].

## Limitations of the study

The main limitation of this study is that it is based on information on malaria research proposals submitted to only one institutional REC, the IRB of the Faculty of Tropical Medicine, Mahidol University, Thailand. It may therefore not be representative of RECs elsewhere in Thailand or around the world to which malaria proposals are submitted. This study did not aim to compare the consent process described in the proposals against any specific guideline, but rather summarized the ethical issues related to a typical standard set of ICP elements used by the FTM-EC when reviewing research proposals. Another important limitation of this study was that the analysis of ICP elements was based on document review, including primarily the notifications to the proposal submitters; subsequently all proposals were reexamined to confirm or extract additional quotations. It should also be noted, however, that most of the authors are/were ethics committee members in FTM-EC panels who had read and/or made comments on such ICP issues from the submitted proposals.

## Conclusions

The results of this study reflect a REC assessment of the informed consent process, as described in proposals submitted. Although all researchers proposed to communicate with their study participants, about two-thirds of them need to improve explanations of study procedures, including study activities, and the specimen/data collection process for their study participants. About 40% of the proposals attracted comments on risk and discomfort, vulnerable status, and compensation. Studies that planned to collect or use new/linked specimens raised more ICP issues than studies using linked data or records. Similarly, studies that involved vulnerable populations raised more ICP issues than those that did not. The REC usually asked researchers to clarify, elaborate, revise, or paraphrase the ICP elements that were deemed to involve inadequate information exchange between researcher and study participant.

In conclusion, many critical ICP elements in malaria research proposals were found to be defective and/or incomplete, and required elaboration or revision before approval could be granted by the REC. Although the findings are based on just one REC, the nature of the study designs, procedures/activities to be performed at the clinic/laboratory, study population and environmental settings for malaria research might be similar elsewhere, and the ICP elements reviewed by RECs are generally the same. The information and analysis of ICP elements in this study could therefore inform the process of preparing proposals for malaria research elsewhere. Being aware of which ICP issues usually arouse concern, and in which types of study designs, would help malaria researchers to plan their studies better, while reducing the number of ethical issues raised by RECs. This would shorten the time required for proposal approval, reduce tension between researchers and the REC, and help researchers to communicate more effectively and ethically with study participants.

## Abbreviations

FTM: Faculty of Tropical Medicine
FTM-EC: Faculty of Tropical Medicine Ethics Committee
FWA: Federal-wide Assurance
IAF: Informed Assent Form
ICF: Informed Consent Form
ICP: Informed Consent Process
IRB: Institutional Review Board
ORS: Office of Research Services
PIS: Participant Information Sheet
REC: Research Ethics Committee
